# AIRWAY POSITIVE PRESSURE VS. EXERCISES WITH INSPIRATORY LOADING FOCUSED ON PULMONARY AND RESPIRATORY MUSCULAR FUNCTIONS IN THE POSTOPERATIVE PERIOD OF BARIATRIC SURGERY

**DOI:** 10.1590/0102-672020180001e1363

**Published:** 2018-07-02

**Authors:** Maura Rigoldi Simões da ROCHA, Stefane SOUZA, Carolina Moraes da COSTA, Daniela Faleiros Bertelli MERINO, Maria Imaculada de Lima MONTEBELO, Irineu RASERA-JÚNIOR, Eli Maria PAZZIANOTTO-FORTI

**Affiliations:** 1 Universidade Metodista de Piracicaba; 2 Clínica Bariátrica de Piracicaba, Piracicaba, SP, Brazil

**Keywords:** Atelectasis, Respiratory exercises, Physiotherapy, Gastroplasty, Obesity, Atelectasia, Exercícios respiratórios, Fisioterapia, Gastroplastia, Obesidade

## Abstract

***Background*:**

Bariatric surgery can trigger postoperative pulmonary complications due to factors inherent to the procedure, mainly due to diaphragmatic dysfunction.

***Aim*:**

To evaluate and compare the effects of two levels of positive pressure and exercises with inspiratory load on lung function, inspiratory muscle strength and respiratory muscle resistance, and the prevalence of atelectasis after gastroplasty.

***Methods*:**

Clinical, randomized and blind trial, with subjects submitted to bariatric surgery, allocated to two groups: positive pressure group, who received positive pressure at two levels during one hour and conventional respiratory physiotherapy and inspiratory load group, who performed exercises with load linear inspiratory pressure, six sets of 15 repetitions, in addition to conventional respiratory physiotherapy, both of which were applied twice in the immediate postoperative period and three times a day on the first postoperative day. Spirometry was performed for pulmonary function analysis, nasal inspiratory pressure for inspiratory muscle strength and incremental test of respiratory muscle resistance for sustained maximal inspiratory pressure, both preoperatively and on hospital discharge on the second postoperative day.

***Results*:**

There was no significant difference (p> 0.05) in the expiratory reserve volume and in the tidal volume in the pre and postoperative periods when compared intra and intergroup. There was no significant difference (p>0.05) in the nasal inspiratory pressure and the maximal inspiratory pressure maintained in the inspiratory load group in the intragroup evaluation, but with a significant difference (p<0.05) compared to the positive pressure group. The prevalence of atelectasis was 5% in both groups with no significant difference (p>0.05) between them.

***Conclusion*:**

Both groups, associated with conventional respiratory physiotherapy, preserved expiratory reserve volume and tidal volume and had a low atelectasis rate. The inspiratory loading group still maintained inspiratory muscle strength and resistance of respiratory muscles.

## RESUMO

*****Racional*** ****:**

A cirurgia bariátrica pode desencadear complicações pulmonares no pós-operatório devido a fatores inerentes ao procedimento, sobretudo pela disfunção diafragmática.

*****Objetivo*** ****:**

Avaliar e comparar os efeitos da aplicação de dois níveis de pressão positiva e dos exercícios com carga inspiratória na função pulmonar, força muscular inspiratória e resistência muscular respiratória e na prevalência de atelectasia após gastroplastia.

*****Métodos*** ****:**

Ensaio clínico, randomizado e cego, com sujeitos submetidos à cirurgia bariátrica, alocados em dois grupos: grupo pressão positiva, que recebeu pressão positiva em dois níveis, durante uma hora e fisioterapia respiratória convencional e grupo carga inspiratória, que realizou exercícios com carga linear pressórica inspiratória, seis séries de 15 repetições, além da fisioterapia respiratória convencional, sendo ambos aplicados duas vezes no pós-operatório imediato e três vezes ao dia no primeiro dia de pós-operatório. Foram realizados espirometria para análise da função pulmonar, pressão inspiratória nasal para força muscular inspiratória e teste incremental de resistência muscular respiratória para pressão inspiratória máxima sustentada, no pré-operatório e no dia da alta hospitalar, no segundo dia de pós-operatório.

*****Resultados*** ****:**

Não houve diferença significativa (p>0,05) no volume de reserva expiratório e no volume corrente no pré e no pós-operatório quando comparados intra e intergrupo. Não houve diferença significativa (p>0,05) na pressão inspiratória nasal e na pressão inspiratória máxima sustentada no grupo carga inspiratória na avaliação intragrupo, mas com diferença significativa (p<0,05) comparada ao grupo pressão positiva. A prevalência de atelectasias foi de 5% em ambos os grupos sem diferença significativa (p>0,05) entre eles.

*****Conclusão*** ****:**

Ambos os grupos, associados à fisioterapia respiratória convencional, preservaram o volume de reserva expiratório e o volume corrente e apresentaram baixo índice de atelectasias. O grupo carga inspiratória ainda manteve a força muscular inspiratória e a resistência dos músculos respiratórios.

## INTRODUCTION

Bariatric surgery is currently considered the most effective treatment for the control and treatment of morbid obesity^14^. However, due to the factors associated with this intervention, such as anesthesia, manipulation of the viscera, loss of muscle integrity due to incision, mainly by laparotomy and consequent pain, lead to diaphragmatic paresis^4^ and restrictive pulmonary behavior^8^, with decreasing volumes and pulmonary and respiratory muscle strength in the postoperative period^24^ ^,^ ^31^.

Thus, the association of these factors contributes to the occurrence of pulmonary complications, being the main causes of morbidity and mortality, increased hospitalization time and hospital cost^18^.

In this sense, the use of different physiotherapy resources, including positive pressure, which promotes pulmonary function restoration and equipment with inspiratory resistive load that also aid in the recovery of pulmonary flows and volumes, through increased respiratory muscle strength and endurance^9^, favors the reduction of atelectasis, pneumonia and time of hospitalization^17^.

However, due to the application of positive pressure, especially in two levels (BIPAP), to decrease diaphragmatic activity through partial muscular rest in obese patients^9^, the hypothesis of this study was that the use of equipment with linear inspiratory pressure load, restoring the inspiratory muscle force, could contribute more effectively to the attenuation of the effects of diaphragmatic dysfunction present in the postoperative period of bariatric surgery.

Therefore, the objective of this study was to evaluate and compare the effects of positive airway pressure and exercises with inspiratory load on lung function, inspiratory muscle strength, respiratory muscle resistance and pulmonary complications in morbidly obese patients after bariatric surgery.

## METHODS

It is a prospective, randomized, blind clinical trial. The study was conducted according to Resolution 466/12 of the National Health Council and approved by the Research Ethics Committee of the Methodist University of Piracicaba (UNIMEP) no. 89/12, and the enrolled subjects signed a free and informed consent form. It was registered in Clinical Trials platform number NCT02682771.

Women aged 25-55 years with a BMI≥40 kg/m^2^ and <55 kg/m^2^, who underwent Roux-en-Y gastric bypass by laparotomy, were included. They should have normal prior spirometry and chest X-rays, non-smokers, no history of chronic lung diseases, no obstructive sleep apnea syndrome, or requiring prior positive airway pressure. Exclusion criteria were hemodynamic instability in the postoperative period, with surgical complications, remaining more than three days in the hospital (outside the protocol of the surgical team), who refused to participate in the study until its completion or inability to evaluation.

Once the inclusion criteria were met, the volunteers underwent a pre-operative evaluation, considered as baseline. After that, randomization by lottery was conducted, where they were allocated in two groups: Positive Pressure Group (GPP), which performed positive pressure in two levels associated with conventional respiratory physiotherapy (FRC) and Group Load Inspiratory (GCI), which performed inspiratory load exercises in addition to the FRC.

**FIGURE 1:**
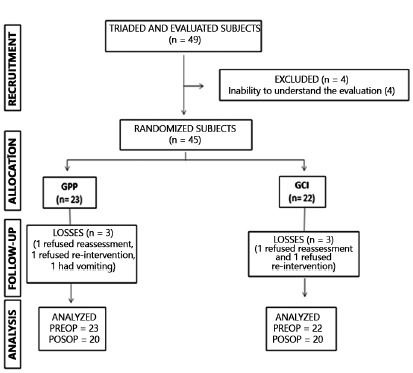
Flowchart of the study casuistry, according to the CONSORT

Of the 49 volunteers evaluated, four were excluded from the study and 45 were randomized, five were not reevaluated and were integrated into the intention-to-treat study, totaling 40 at the end (Figure 1).

n=number of subjects in the group; GPP=positive pressure group; GCI=inspiratory loading group; PREOP=preoperative; POSOP=postoperative

### Evaluation

All volunteers were assessed preoperatively and on hospital discharge on the second postoperative day, and the researchers were blinded according to their assignments, evaluations, interventions, data treatment, as well as to two radiological examinations.

In the postoperative evaluation, a visual analogue scale was used to classify the pain10 in order to minimize its interference. If the referred pain intensity was greater than or equal to 4, analgesics were given as prescribed by the physician. The evaluation was started after 30 min if the pain intensity was less than or equal to 3.

### Anthropometric data

Body mass and height were obtained through a digital scale, and the body mass index (BMI) was calculated by means of the equation: body mass (Kg) / stature^2^ (m).

### Pulmonary function

A computerized ultrasound spirometer with flow sensor (Microquark; Cosmed, Rome, Italy) was used to evaluate lung volumes, flows and capacities, following the norms recommended by the American Thoracic Society^1^ and the guidelines for lung function tests^25^. Slow vital capacity maneuvers (SVC), forced vital capacity (FVC) and maximal voluntary ventilation (VVM) were performed, with the highest values ​​of the variables being computed.

### Inspiratory muscle strength and maximal inspiratory pressure (MIP)

It was performed with the purpose of prescribing the inspiratory load of the inspiratory powerbreathe K3^®^, by means of the MVD 300^®^ digital manovacuometer (GlobalMed, RS, Brazil), from the residual volume (VR)^7^, with sustained effort by at least 2 s. At least five maximal inspiratory efforts, technically acceptable and reproducible, with values ​​close to each other (≤10%) were requested. For the analysis of the data, the highest value was recorded^22^.

### Nasal inspiratory pressure (PIN)

For this measurement, the Sniff technique, generated by a nasal pressure peak from the functional residual capacity (CRF), was used through a silicone nasal plug connected to a MVD 300^®^ digital manovacuometer (GlobalMed, RS, Brazil). Ten maneuvers were requested^21^, with an interval of 30 s between each one, using as the criterion for selection of the acceptable Sniff, generation of the highest pressure peak and duration between 0-5 s^30^. It is a validated technique, with accuracy and high correlation with MIP, being considered its more physiological execution^27^ besides promoting greater comfort, benefits that should be considered in postoperative patients^15^.

### Resistance of respiratory muscles and maximal sustained inspiratory pressure (PImaxS)

This test was performed using Powerbreathe K3^®^ equipment (Gaiam Ltd, Southam, Warwickshire, UK), and volunteers should generate strong, deep inspirations followed by complete expirations. At the end of the expiration, after the cessation of flow, an acoustic signal from the device was emitted, signaling the beginning of a new respiratory cycle^19^. The test started with 30% of MIP^2^, and a load of 10 cm H_2_O was added to each phase of 30 respiratory cycles. After the end of each stage, the volunteer remained in rest for 1 min to restart it. The test was interrupted when the predetermined pressure for three consecutive breaths was not reached or if there was a dyspnea symptom, with the MIP value being the highest sustained load for at least 15 breaths.

This device has the differential to load digitally adjustment of the and provides the data of power, volume and training index (IT), which aid in the evaluation of the behavior of respiratory muscles. These data reflect, respectively, the energy of the inspirations, the respiratory pattern and the inspiratory work generated^19^.

### Chest X-ray

Thoracic radiographs were performed in the posteroanterior incidence, with the subjects in the orthostatic position, on the day of discharge, and atelectasis were the reports that mentioned the words “atelectasis”, “pulmonary hypoexpansion” or “hypoexpansion of pulmonary field(s)”, regardless of location and size.

### Intervention

#### *Positive pressure group*

This group received non-invasive positive pressure on two levels via the BIPAP Synchrony II-Phillips-Respironics^®^ (Murrysville, Pennsylvania, USA) face mask. The inspiratory positive pressure (IPAP) was initially adjusted to 12 cmH_2_O and readjusted according to tolerance, maintaining respiratory rate (RR) below 30 breaths per minute (rpm) and tidal volume around 8-10 ml/kg. Positive airway expiratory pressure (EPAP) was set at 8 cmH_2_O, both adjustments being determined from the Brazilian Recommendations on Mechanical Ventilation^5^. The volunteers remained with the device for one hour, immediately after the return of the anesthetic recovery room and after 4 h and, on the first postoperative day, three times a day, with interval of 6 h between the sessions.

#### *Inspiratory loading group*

This group performed inspiratory load exercises using the Powerbreathe K3^®^ equipment, being applied at the same frequency as the previous group. 40% of the MIP^4^ value measured in the preoperative period was used as resistance. The volunteer was instructed to inhale to overcome the resistance of the device and later to perform normal expiration. Six series were performed with 15 repetitions each, with a range of 30-60 s between sets.

Both groups also performed CRF, which consisted of diaphragmatic breathing exercises, deep and fractional inspirations, respiratory exercises associated with upper limb movement and use of incentive spirometry, with a series of 10 repetitions each exercise, in addition to ambulation^12^.

### Statistical analysis

Data were analyzed using SPSS software version 17.0. For the normal distribution of the data the Shapiro-Wilk test was used. Student’s t-test or Mann-Whitney test was applied for the comparison of anthropometric and age characteristics and for intergroup analysis, using the values ​​of the differences between the pre and postoperative periods. For the intragroup analysis, comparing the pre and postoperative, Student’s or Wilcoxon’s T tests were used. Fischer’s exact test was performed to assess the prevalence of atelectasis in each group, and a significance level of 5% was adopted for all analyzes.

## RESULTS

### Age and anthropometric characteristics

The mean age was 38.2±9.40 for GPP and 36.9±5.92 for GCI, and BMI of 46.94±4.54 for GPP and 44.66±4.06 for GCI, with no differences between groups for these variables (p>0.05).

### Pulmonary function

There was a significant reduction of variables, except for VRE and VC in both groups (Table 1), but with no significant difference between groups (p>0.05).

**TABLE 1:** Comparison of the measures of the spirometric variables in absolute values and percentages of the predicted of the Slow Vital Capacity maneuver for each group in the pre and postoperative periods, expressed as mean and standard deviation

		GPP (n=23)	GCI (n=22)
		Pre Post	Pre Post
CVL (L) p	M DP	3.06 2.44 ±0.58 ± 0.38 0.005*	3.14 2.62 ±0.42 ±0.44 < 0.001*
CVL (% prev) p	M DP	94.30 75.80 ±8.52 ± 8.78 0.005*	94.95 79.55 ±8.24 ±12.26 < 0.001*
VRE (L) p	M DP	0.50 0.45 ±0.22 ±0.24 0.2	0.55 0.45 ±0.23 ±0.25 0.1
VRE (% prev) p	M DP	44.00 38.75 ±16.16 ±17.34 0.1	47.75 39.40 ±17.94 ±21.13 0.1
VRI (L) p	M DP	1.97 1.46 ±0.50 ±0.30 < 0.001*	2.14 1.68 ±0.31 ±0.26 < 0.001*
VC (L) p	M DP	0.76 0.72 ±0.17 ±0.20 0.5	0.73 0.69 ±0.14 ±0.22 0.4

Pre=preoperative; post=postoperative; n=number of subjects; GPP=positive pressure group; GCI=inspiratory loading group; CVL=slow vital capacity; VRE=expiratory reserve volume; VRI=inspiratory reserve volume; VC=tidal volume; % prev=percentage of predicted; L=liter; M=average; SD=standard deviation; *=significant difference between pre and postoperative=p<0.05

In relation to forced vital capacity (FVC) and its consequences, there was a significant difference (p<0.05) with reduction of all postoperative values in both groups, but without significant difference between them (p>0.05).

Regarding maximum voluntary ventilation (VVM), despite the significant difference (p<0.05), which was a reduction in relation to the preoperative period, in both groups postoperative values were above 80% of predicted, that is, maintaining a normality pattern (Table 2). There was no significant difference between groups (p=0.08).

### Inspiratory muscle strength and respiratory muscle resistance

Table 3 shows a significant reduction of PIN, PImaxS and their unfolding, except IT in GPP, with maintenance of these values, except volume in the GCI.

**TABLE 2:** VVM values for each group in the pre and postoperative periods, expressed in absolute values and predicted percentages, in mean and standard deviation.

		GPP (n=23)	GCI (n=22)
		Pre Post	Pre Post
VVM (L) p	M DP	108.61 84.21 ±17.29 ± 11.15 0.001	107.45 92.69 ±14.83 ±17.52 0.01
VVM (% prev) p	M DP	103.65 81.00 ±13.24 ±12.52 0.001	101.15 87.25 ±12.21 ±14.89 0.01

Pre=preoperative; post=postoperative; n=number of subjects; L=liter; GPP=positive pressure group; GCI=inspiratory loading group; VVM=maximal voluntary ventilation; % prev=percentage of predicted; M=average; SD=standard deviation; significant difference between the pre and postoperative periods=p<0.05

**TABLE 3:** Comparison of the measures of PIN, PImaxS, potency, volume and training index for each group in the pre and postoperative period. Values expressed as mean and standard deviation.

		GPP (n=23)	GCI (n=22)
		Pre Post	Pre Post
PIN (cmH20) p	M DP	86.80 75.75 ±16.35 ±19.80 0.018*	87.15 80.55 ±15.14 ±19.38 0.128
PImáxS (cmH20) p	M DP	38 33.5 ±8.94 ±5.87 0.009*	42 38.5 ±10.56 ±8.13 0.2
POTENCY (W) p	M DP	2.99 2.33 ±1.19 ±1.05 0.03*	3.11 2.65 ±1.23 ±1.31 0.07
VOLUME (L) p	M DP	2.03 1.09 ±3.31 ±0.32 0.04*	1.4 1.0 ±0.43 ± 0.26 0.03*
IT (%prev) p	M DP	66.2 56.5 ±31.4 ±40.1 0.3	80.65 78.1 ±18.0 ±27.51 0.7

GPP=positive pressure group; GCI=inspiratory loading group; n=number of subjects; pre=preoperative; post=postoperative; PIN=nasal inspiratory pressure; CmH_2_0=centimeters of water; PImaxS=sustained maximum inspiratory pressure; W=watt; L=liter; IT=training index; % prev=percentage of predicted; M=average; SD=standard deviation; * significant difference between the pre and postoperative periods=p<0.05

Comparing these values between the groups, there was a significant difference (p=0.04) in PIN, demonstrating GCI superiority in maintaining this value in relation to GPG.

The prevalence of atelectasis in the respective groups was 5% in both groups, with no significant difference between them (p=1).

## DISCUSSION

The present study demonstrated the maintenance of the spirometric values ​​of VRE and VC in both groups, and the ICG still managed to maintain the PIN and PImaxS.

These findings are of great relevance, since pulmonary mechanics is altered postoperatively, with an increased risk of atelectasis^34^.

A review by Delgado and Lunardi^10^ showed that the main and most frequent respiratory change in the postoperative period of bariatric surgery was spirometry, with the reduction in CV being the most reported.

Regarding VRE, morbidly obese individuals present, independently of having undergone abdominal surgery, a reduction in this value when compared to non-obese^20^, being the most frequent finding in these individuals^28^. It is known that its preservation in the postoperative period may contribute to a decrease in atelectasis during this period, as observed by Baltieri et al.^4^, who applied non-invasive positive pressure in two levels for 1 h after the end of bariatric surgery, evidenced restoration of VRE and reduction of the prevalence of atelectasis.

Such benefits are promoted by the application of positive pressure at two levels by the combination of inspiratory pressure with positive pressure at the end of expiration, allowing recruitment of the collapsed alveolar zones, thereby improving pulmonary function^30^.

In relation to the inspiratory load devices, Westerdahl et al.^33^ proposed that the more potent the muscle contraction, due to stronger the respiratory muscle promoted by them, greater is the transpulmonary pressure gradient generated, thus mobilizingmore air volume^29^.

Postoperatively, in addition to the spirometric reduction, there is a decrease in MIP, which reflects the diaphragmatic dysfunction^13^, also observed in this study, due to the decrease in PIN, identifying the muscular inefficiency promoted by the factors inherent to the surgical procedure, previously described.

In the study by Casali et al.^9^, despite the significant reduction in respiratory muscle strength on the second postoperative day, the group that performed exercises with inspiratory load showed a return of baseline values ​​of inspiratory muscle strength earlier than the control group. Both groups were followed up until the 30^th^ postoperative day, with an 8% loss in the MIP of the control group, whereas in the group that carried inspiratory muscle load, there was a gain of 13%. In the present study, the ICG was able to maintain the PIN on the second postoperative day.

Regarding respiratory muscle resistance, it reflects the ability of the muscle to support loads, which may be increased in situations that imply greater demands, such as respiratory complications.

The exercise program with inspiratory load used as a proposal for intervention in the present study should not be understood as inspiratory muscle training, since the time of accomplishment was short, and it is not possible during this period, a change in the type of muscle fibers. However, it is suggested that the preservation of respiratory muscle resistance, evidenced in this study by the maintenance of MIPs and normal values ​​of VVM (predicted %) in the ICG, may contribute to the reduction of dyspnea and exercise tolerance, as found by Villiot -Danger et al.^32^ and, consequently, in the prevention of pulmonary complications.

For PImaxS and its consequences, there was a significant reduction in GPP, except for IT, in addition to the normal value of VVM (predicted %), which could be justified by the improvement of pulmonary mechanics promoted by positive pressure, favoring a better performance of the respiratory musculature. Regarding the maintenance of these variables, except volume in the GCI, it can be concluded that the inspiratory load exercises with the PowerBreathe^®^ promoted maintenance of muscular performance, combining strength and speed, but were not able to maintain the amount of air inspired during the incremental test.

The low prevalence of atelectasis, 5% for both groups, is possibly due to the maintenance of VRE, volume associated with CRF, which may have favored greater pulmonary stabilization. It is emphasized that all atelectases were subclinical, not promoting any functional impact. This result is relevant, since according to Baltieri et al.^3^, the incidence of atelectasis in the postoperative period of bariatric surgery reaches 37.8%. In addition, no other pulmonary impairment was found in the analysis of chest radiographs, being justified by the early onset and number of sessions performed in the physiotherapeutic care. Thus, it is suggested that interventions in both groups be effective, demonstrating the importance of physiotherapy in the prevention of pulmonary complications^23^.

## CONCLUSION

Both two-level positive pressure application and the inspiratory load exercises associated with conventional respiratory physiotherapy were beneficial in preserving important pulmonary volumes in the prevention of pulmonary complications, as evidenced by the low atelectasis rate. It´s highlighted thatthe results promoted by the exercises with inspiratory load, which demonstrated superiority in maintaining inspiratory muscle strength and respiratory muscle resistance, suggesting a greater attenuation of the effects of diaphragmatic dysfunction present in the postoperative period of bariatric surgery.
